# Venetoclax Plus Azacitidine as a Bridge Treatment to Allogeneic Stem Cell Transplantation in Unfit Patients with Acute Myeloid Leukemia

**DOI:** 10.3390/cancers16061082

**Published:** 2024-03-07

**Authors:** Tzu-Ting Chen, Ching-Chan Lin, Wen-Jyi Lo, Ching-Yun Hsieh, Ming-Yu Lein, Che-Hung Lin, Chen-Yuan Lin, Li-Yuan Bai, Chang-Fang Chiu, Su-Peng Yeh

**Affiliations:** 1 Division of Hematology and Oncology, Department of Internal Medicine, China Medical University Hospital, Taichung 404, Taiwan; little_zi2000@yahoo.com.tw (T.-T.C.); linchin13256@gmail.com (C.-C.L.); tsienkoala@yahoo.com.tw (C.-Y.H.); 012604@caaumed.org.tw (M.-Y.L.); windsun@gmail.com (C.-H.L.); jnynlin@gmail.com (C.-Y.L.); lybai6@gmail.com (L.-Y.B.); 2 Division of Hematology and Oncology, Department of Internal Medicine, An Nan Hospital, Tainan 700, Taiwan; 3 College of Medicine, China Medical University, Taichung 404, Taiwan; wenjlo@gmail.com; 4 Stem Cell Research Laboratory, Department of Medical Research, China Medical University Hospital, Taichung 404, Taiwan; 005686@tool.caaumed.org.tw

**Keywords:** acute myeloid leukemia, venetoclax, azacytidine, allogeneic stem cell transplantation

## Abstract

**Simple Summary:**

The study explores a promising treatment avenue for acute myeloid leukemia (AML), emphasizing the drug combination venetoclax and azacitidine (VEN plus AZA) to improve patient survival and enable stem cell transplantation in those unsuitable for intensive chemotherapy. Comparative analysis with conventional treatment shows comparable survival rates, influenced by genetic factors. In conclusion, for older AML patients unable to endure intensive chemotherapy, the VEN plus AZA regimen emerges as a viable, less taxing option, offering the potential for curative stem cell transplantation.

**Abstract:**

Background: Allogeneic hematopoietic stem cell transplantation (HSCT) is rarely recommended for unfit patients with newly diagnosed acute myeloid leukemia (AML). Patient survival can improve with venetoclax plus azacitidine (VEN plus AZA). However, the long-term outcome of this treatment strategy is still unsatisfactory. The high response and low treatment toxicity rates of patients receiving VEN plus AZA can provide an opportunity for HSCT among unfit patients. Nevertheless, the outcomes and complications of VEN plus AZA, followed by HSCT, remain unclear. Methods: This single-center retrospective study aimed to compare patients with newly diagnosed AML receiving VEN plus AZA as induction therapy (*n* = 27) to those receiving the conventional I3A7 regimen as induction therapy (*n* = 34). Result: The 1-year overall survival, relapse, and non-relapse mortality rates in the two groups were similar. The cytogenetic risks and the hematopoietic cell transplantation-specific comorbidity index are the most significant predictive factors of overall survival. Conclusion: In older patients unfit for intensive chemotherapy, a low-intensity regimen with VEN plus AZA is a suitable bridge therapy. Furthermore, allo-HSCT is feasible and can be a curative option.

## 1. Introduction

Intensive induction chemotherapy, followed by allogeneic hematopoietic stem cell transplantation (allo-HSCT), is a treatment option for patients with newly diagnosed high-risk acute myeloid leukemia (AML). The complete remission rates associated with standard induction chemotherapy comprising cytarabine and anthracycline (7 + 3) range from 70% to 80% [1,2]. However, the response rate decreased to 40–60% in patients aged >60 years [3]. Induction chemotherapy is associated with significant morbidity and mortality in elderly patients with comorbidities. The early mortality rates are 25–30% in patients aged 60–69 years and >50% in those aged ≥ 70 years [4].

Venetoclax, a potent BCL2 inhibitor, exhibits apoptotic effects on BCL2-dependent malignant cells, as demonstrated in preclinical studies. In acute myeloid leukemia, the efficacy of single-agent venetoclax is modest, indicating the need for combinational approaches. The combination of venetoclax and azacitidine proves synergistic in AML, involving the down-regulation of myeloid-cell leukemia 1 (MCL1) and the induction of pro-death proteins NOXA and PUMA. This combination inhibits prosurvival proteins MCL1 and BCL-XL, intensifying leukemia cells’ reliance on BCL2. Notably, azacitidine and venetoclax jointly induce cell death in AML-derived cell lines, providing promising prospects for therapeutic intervention. Venetoclax (VEN) combined with azacitidine (AZA), a hypomethylating agent, is associated with better response and survival in patients with comorbidities or those aged >65 years [5,6]. Although the outcomes of VEN plus AZA have improved, this combination therapy is not considered a treatment option for high-risk AML [6,7]. However, VEN plus AZA has a tolerable safety profile and a low 30-day mortality rate. Hence, it provides an opportunity for patients to recover from their exhausted disease status and bridge to allo-HSCT [6,7].

Several retrospective reports have described the successful use of this regimen as a bridge to allo-HSCT in elderly patients with AML [8,9,10,11,12]. However, there is a paucity of objective assessments of the mortality risk of the so-called unfit population, and all known studies used matched unrelated donors or umbilical cord blood as their major donor sources. Therefore, the post-transplantation outcomes of patients receiving haploidentical donors are unrepresented.

This study aimed to evaluate the outcomes of allo-HSCT in patients with newly diagnosed AML who were unfit for 3 + 7 induction chemotherapy and received VEN plus AZA as induction therapy. Particularly, >50% of the patients in our cohort underwent post-transplant cyclophosphamide-based haploidentical peripheral stem cell transplantation.

## 2. Materials and Methods

### 2.1. Patient Population

This retrospective study was approved by the Institutional Review Board of China Medical University Hospital (CMUH) under approval number DMR-107-019. We enrolled patients who were newly diagnosed with AML and underwent HSCT after remission following induction therapy, aligning with ELN criteria [3]. The standard induction therapy (two cycles of the I3A7 regimen) at CMUH. Since 2018, Two cycles of VEN plus AZA have been administered to patients ineligible for the I3A7 regimen (due to comorbidities or senescence). Unfit AML patients were considered ineligible for standard induction therapy if they were 75 years of age or older or if they had at least one of the following coexisting conditions precluding intensive chemotherapy: a history of congestive heart failure for which treatment was warranted or an ejection fraction of 50% or less or chronic stable angina, a diffusing capacity of the lung for carbon monoxide of 65% or less or a forced expiratory volume in 1 s of 65% or less, and an Eastern Cooperative Oncology Group performance-status score of 2 or 3 (on a 5-point scale, with higher numbers indicating greater disability) at the time of diagnosis. Therefore, patients who received VEN plus AZA as the first-line therapy, followed by HSCT, were assigned to a different cohort.

### 2.2. Transplantation Protocol

The myeloablative conditioning regimen comprised 3.2 mg/kg/day of intravenous busulfan for 4 days and 40 mg/m^2^/day of intravenous fludarabine for 4 days. The reducing intensity conditioning regimen comprised 3.2 mg/kg/day of intravenous busulfan for 2 days and 40 mg/m^2^/day of fludarabine for 4 days.

The prophylactic treatment against graft-versus-host disease (GVHD) comprised 4.5 mg/kg of rabbit ATG (Thymoglobuline, Genzyme, Cambridge, MA, USA) on days 2–4, cyclosporine since day 1, and mycophenolate sodium (720 mg twice daily) from day 0 after stem cell infusion to day 35. Intravenous cyclosporine (1.5 mg/kg) was administered twice daily to maintain the trough level between 200 and 400 ng/mL. Overall, 50 mg/kg of cyclophosphamide was administered on days 3 and 4 post-haploidentical HSCT.

### 2.3. Measurable Residual Disease Assessment and Definition

We utilized EuroFlow 8-color antibody panels, which included the integration of diagnostic leukemia-associated immunophenotype (LAIP) and a different from normal (DfN) aberrant immunophenotype strategy for MRD detection. The core MRD markers were CD34, CD117, CD45, CD33, CD13, CD56, CD7, and HLA-DR, and the cut-off level for MRD positivity was 0.1%. The measurable residual disease (MRD) status was assessed with multiparametric flow cytometry at the pre-HSCT bone marrow time point and at any available post-HSCT bone marrow time point. Post-transplant disease assessments were routinely conducted on days 30, 90, and 180, and relapse was suspected at any time.

### 2.4. Outcome Definitions

The cytogenetic risk stratification of AML was based on the ELN classification [3]. Further, each newly diagnosed patient was routinely examined for FMS-like tyrosine kinase-3 internal tandem duplication (Flt3 mutation). However, the examination of other molecular genes (including *NPM1*, *CEBPA*, *WT1*, *RUNX1*, *TET2*, and *IDH1*) was not routinely performed. The hematopoietic cell transplantation-specific comorbidity index (HCT-confidence interval (CI)) was calculated as described in a previous study [13]. Per the ELN criteria, the disease status before transplantation was defined as follows: complete remission (CR)—bone marrow blasts of <5%, the absence of circulating blasts and blasts with Auer rods, the absence of extramedullary disease, absolute neutrophil counts of ≥1000/µL, and platelet count ≥100,000/µL; CR with incomplete hematologic recovery—all CR criteria except for residual neutropenia (<1000/µL) or thrombocytopenia (<100,000/µL); and morphologic leukemia-free state—a bone marrow blasts percentage of <5%, the absence of blasts with Auer rods and extramedullary disease, and without the required hematologic recovery. Patients with a bone marrow myeloblast percentage of >5% were diagnosed with refractory disease [3].

Infectious diseases were identified from the time of HSCT until the relapse of the underlying hematologic cancer or death. The clinical diagnosis and grading of graft-versus-host disease (GVHD) followed established protocols. For acute GVHD, the Consensus Conference on Acute GVHD criteria, specifically the grading consensus criteria [14,15], were utilized. In addition, the National Institutes of Health consensus criteria for chronic GVHD were employed for the diagnosis and grading of chronic GVHD. [16,17].

### 2.5. Statistical Analysis

The study’s endpoints, which were measured from transplantation to the events of interest, included overall survival (OS), cumulative incidence of disease relapse, non-relapse mortality (NRM), acute GVHD, and chronic GVHD. If no events occurred, individuals were censored at the last follow-up date. An event for OS is defined as death from any cause. NRM was defined as death without relapse. Disease relapse and NRM were considered competing-risk events during cumulative incidence calculations. The CI of acute or chronic GVHD was analyzed, with relapse and death without GVHD as competing risks. Cox proportional hazard models were used to calculate hazard ratios in multivariate analyses. The Fine–Gray subdistribution hazard model was employed in multivariate analyses in the presence of competing events [18]. Forest plots were created to summarize the multivariate Cox proportional hazard model results and their respective hazard ratios and confidence intervals. A *p*-value of <0.05 was considered statistically significant. All analyses were performed using R version 4.2.2.

## 3. Results

### 3.1. General Characteristics of the Participants

The median follow-up period of the whole cohort was 14.4 (range: 0.43–59.3) months. The median follow-up times of patients who received induction treatment with chemotherapy (chemotherapy group) and those who were considered unfit for intensive chemotherapy during AML diagnosis and who received induction treatment with VEN plus AZA (VEN plus AZA group) were 14.8 and 14.4 months, respectively. The general characteristics of this study’s participants are shown in Table 1. Overall, 34 patients were included in the chemotherapy group and 27 in the VEN-plus-AZA group. The 4-week mortality rates of patients treated with intensive chemotherapy were predicted via assessments of age, frailty, organ dysfunction, cytogenetic abnormality, and infection, per Koji Sasaki et al. [19]. A predicted 4-week mortality rate of 20% was considered an acceptable risk for intensive chemotherapy. There were 8 (29.6%) out of 27 high-risk patients in the VEN-plus-AZA group. Only 3 (8.8%) out of 34 patients in the chemotherapy group were at high risk of early mortality while receiving intensive chemotherapy (*p*-value = 0.048). 

The sex distributions of the two groups did not differ significantly from each other. The VEN-plus-AZA group had a significantly higher median age than the chemotherapy group (median: 65 (48–78) vs. 40 (19–68) years, *p*-value < 0.005). The VEN-plus-AZA group had a significantly higher number of MDS-related AML cases than the chemotherapy group (11.8% vs. 55.6%, *p*-value < 0.005). The distributions of cytogenetic risks and Flt3 mutation status between the two groups did not differ significantly.

The percentages of flow-based MRD did not significantly differ between the VEN plus AZA and chemotherapy groups (77.8% vs. 81.5%, *p*-value = 1). The VEN-plus-AZA group had a higher number of patients with a high HCT-CI than the chemotherapy group (51.9% vs. 20.6%, *p*-value = 0.02). Additionally, the patients in the VEN-plus-AZA group were less likely to receive myeloablative conditioning regimens. Approximately 59.3% of patients in the VEN-plus-AZA group and 50% of those in the chemotherapy group received haploidentical peripheral stem cell transplantation.

### 3.2. Engraftment and Infection

All patients in the chemotherapy group achieved neutrophil and platelet engraftment. However, one patient in the VEN-plus-AZA group had primary graft failure. The median number of neutrophil engraftment days in the chemotherapy and VEN-plus-AZA groups was 13 (*p*-value: 0.87). The median number of platelet engraftment days in the chemotherapy and VEN-plus-AZA groups were 11.5 and 12, respectively (*p*-value: 0.89).

As shown in Table 2, there were seven infectious events (*n* = 6, bloodstream bacterial infection, and *n* = 1, viral infection [echovirus]) within 30 days after stem cell infusion in 34 (21%) patients in the chemotherapy group. All bacterial infections were treated successfully with antibiotics; however, one patient with a viral infection progressed to fatal acute respiratory failure syndrome. Eight infectious events occurred within 30 days after stem cell infusion in 27 (30%) patients in the VEN-plus-AZA group. 

In total, 16 (47.06%) patients in the chemotherapy group and 17 (63.0%) in the VEN-plus-AZA group experienced EBV reactivation. In the chemotherapy group, 9 (56.25%), 6 (37.5%), and 1 (6.25%) of the 16 patients with EBV reactivation received haploidentical HSCT, MUD HSCT, and MSD HSCT, respectively. In the VEN-plus-AZA group, 10 (58.8%) and 7 (41.2%) of the 17 patients with EBV reactivation received haploidentical HSCT and MUD HSCT, respectively. Only one patient in each group developed a post-transplant lymphoproliferative disorder, and both patients received haploidentical HSCT.

Prophylactic treatment with letermovir has been routinely administered for haploidentical HSCT since June 2020. Six patients with haploidentical HSCT in the VEN-plus-AZA group and 15 with haploidentical HSCT in the chemotherapy group did not receive letermovir prophylaxis. In terms of CMV infection, 18 (52.94%) of the 34 patients in the chemotherapy group experienced CMV reactivation. However, 14 (51.85%) of the 27 patients in the VEN-plus-AZA group had CMV reactivation (*p*-value = 0.80).

### 3.3. Analysis of OS, Relapse, and NRM

**Figure 1:**
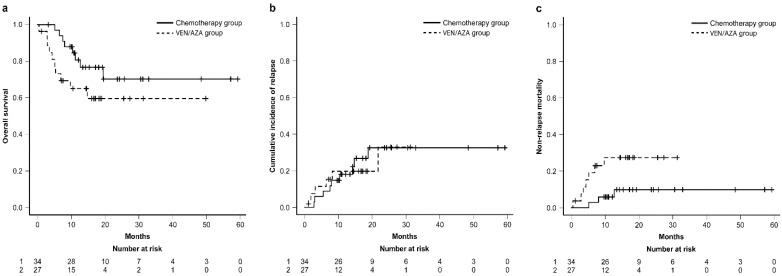
Cumulative incidence and survival analyses according to treatment (VEN/AZA group versus chemotherapy group) (**a**) overall survival; (**b**) cumulative incidence of disease relapse; (**c**) cumulative incidence of non-relapse mortality.

During the follow-up period, a total of eighteen individuals died, with six in the CT group and twelve in the VEN + AZA group. The 1-year OS rates of the VEN plus AZA and chemotherapy groups were 65.3% (95% CI: 43.4–80.1%) and 80.7% (95% CI: 61.7–90.9%), respectively. There was no significant difference in OS between the two groups (*p*-value = 0.13), as presented in Figure 1a. Fifteen individuals experienced relapse, seven in the CT group, and eight in the VEN + AZA group. As shown in Figure 1b, the 1-year CIs of leukemia relapse were 19.7% (95% CI: 6.9–37.3%) in the VEN-plus-AZA group and 18.0% (95% CI: 7.1–32.8%) in the chemotherapy group (*p*-value: 0.91). In total, ten people died due to non-relapse mortality, with three in the CT group and seven in the VEN + AZA group. As depicted in Figure 1c, the 1-year CIs of NRM were 27.4% (95% CI: 11.7–45.8%) in the VEN-plus-AZA group and 5.9% (95% CI: 1.0–17.4%) in the chemotherapy group (*p*-value: 0.053).

We performed a univariate analysis of OS with several factors (including age, HSCT type, AML subtype, cytogenetic risk, HCT-CI, conditioning regimens, response to induction therapy, MRD on flow cytometry, and Flt3 mutation status). The results of this analysis are presented in Table 3. Results showed that only HCT-CI and cytogenetic risk were significant prognostic factors. The 1-year OS rates were 82.00% in patients with an HCT-CI of <2 (95% CI: 65.9–91.0%) and 58.3% in those with an HCT-CI of ≥2 (95% CI: 33.4–76.7%) (*p*-value = 0.02). The patients with good-intermediate cytogenetic risks had a significantly better 1-year OS rate (80.4%, 95% CI: 65.7–89.3%) than those with poor cytogenetic risks (51.1%, 95% CI: 21.6–74.5%) (*p*-value = 0.03).

As shown in Table 3, based on the univariate analysis of disease relapse, the status of the Flt3 mutation and the intensity of the conditioning regimen were found to be significant prognostic factors. The 1-year relapse rates of patients receiving the myeloablative conditioning (MAC) regimen and those receiving the reduced-intensity conditioning regimen were 4.3% (95% CI: 0.3–18.7%) and 27.4% (95% CI: 14.0–42.5%) (*p*-value = 0.04), respectively. The 1-year relapse rates were 14.7% (95% CI: 5.8–27.5%) in patients without the Flt3 mutation and 27.8% (95% CI: 9.6–49.6%) in patients with the Flt3 mutation (*p*-value = 0.02). According to a univariate analysis, young patients were more likely to have a lower NRM, as shown in Table 3. The 1-year NRMs of patients aged <60 years and those aged >60 years were 6.1% (95% CI: 1.0–17.9%) and 26.7% (95% CI: 11.4–44.8%) (*p*-value = 0.07), respectively.

Based on a multivariate analysis of OS, HCT-CI (high vs. low, hazard ratio [HR]: 3.50, 95% CI: 1.14–10.73, *p*-value = 0.03), cytogenetic risk (poor vs. good-intermediate, HR: 7.07, 95% CI: 1.65–30.33, *p*-value < 0.01), and the conditioning regimen (MAC vs. reduced-intensity conditioning regimen, HR: 5.79, 95% CI: 1.02–32.70, *p*-value = 0.05) were significant independent prognostic factors as presented in Supplemental Figure S1. There were no statistically independent prognostic factors based on the multivariate analysis of disease relapse and NRM, as shown in Supplemental Figures S2 and S3. Therefore, the use of VEN plus AZA, or intensive chemotherapies, as induction therapy is not a significant independent prognostic factor for OS, disease relapse, or NRM. 

### 3.4. Incidence of GVHD between the Two Treatment Groups

**Figure 2:**
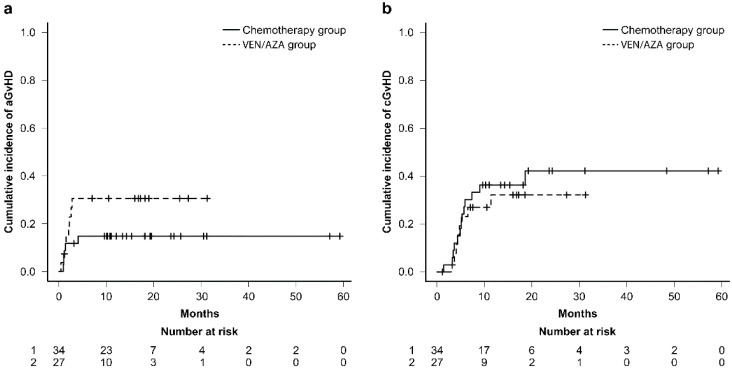
(**a**) Cumulative incidence of grade 2–4 acute GVHD and (**b**) chronic GVHD according to treatment (VEN/AZA group versus chemotherapy group).

Thirteen individuals encountered acute GVHD (aGVHD), with two in the CT group and eleven in the VEN + AZA group. Twenty-one individuals had chronic GVHD (cGVHD), with nine in the CT group and twelve in the VEN + AZA group. The CIs of grades 2–4 acute GVHD within 100 days after transplantation were 11.8% (95% CI: 3.6–25.1%) in the chemotherapy group and 30.6% (95% CI:14.2–48.7%) in the VEN-plus-AZA group. Hence, the incidence of aGVHD did not significantly differ between the two groups (*p*-value = 0.16) as presented in Figure 2A. The CIs of 1-year chronic GVHD (cGVHD) were 36.3% (95% CI: 20.2–52.6%) in the chemotherapy group and 32.2% (95% CI: 14.5–51.3%) in the VEN-plus-AZA group. Therefore, the incidence of cGVHD did not significantly differ between the two groups (*p*-value = 0.61), as shown in Figure 2b. 

Univariate analyses of aGVHD and cGVHD revealed that there were no significant differences in age, HSCT type, AML subtype, cytogenetic risk, HCT-CI, conditioning regimens, response to induction therapy, MRD, or Flt3 status between the two groups, as shown in Table 4. 

A multivariate analysis was also performed, and its results showed that age, HSCT type, AML subtype, cytogenetic risk, HCT-CI, conditioning regimens, response to induction therapy, Flt3 mutation status, and induction treatment were not significant independent prognostic factors of aGVHD and cGVHD as presented in Supplemental Figures S4 and S5.

## 4. Discussion

In this study, we demonstrated that VEN plus AZA is a promising induction treatment for patients with newly diagnosed AML. It is a relatively safe and effective bridge therapy to allo-HSCT in unfit patients who could suffer early mortality when receiving intensive chemotherapies. Post-transplantation outcomes were comparable between patients receiving VEN plus AZA and those receiving conventional intensive chemotherapies.

Conventionally, cytoreduction with intensive induction therapy is mandatory for patients who receive HSCT. However, in older patients or those with comorbidities, intensive chemotherapy is associated with a high risk of early mortality. The application of VEN plus AZA, or low-dose cytarabine, improved the outcomes of unfit patients with newly diagnosed AML based on the findings of phase 3 VIALE-A and VIALE-C trials [6,7]. However, the 2-year OS of patients who received these treatment options was only 30–50% [6,20]. Therefore, they are not considered curative therapies. Nevertheless, HSCT is still required. Due to high response rates and low treatment-related toxicity rates, retrospective studies demonstrated that even patients unfit for standard induction chemotherapy can still have a favorable 1-year post-transplant OS (60–78%) if they proceed to allo-HSCT after receiving VEN-based therapies [12,21]. However, the impact of remission induction therapy with low-intensity regimens such as VEN plus AZA and intensive chemotherapy on post-transplant outcomes has not been evaluated either.

Several retrospective studies have addressed this issue. For example, Pasvolsky et al. compared the outcomes of VEN plus AZA, followed by HSCT, between patients with AML and a historical cohort of patients who received HSCT after intensive chemotherapy. They found that the outcomes were similar for both groups of patients [9]. Winters A C et al. performed a single-center analysis comparing 29 patients receiving HSCT after VEN-plus-AZA treatment and 140 patients receiving intensive chemotherapy in the first remission and found no significant differences in OS, NRM, relapse, aGVHD, and cGVHD between the two groups [8]. In line with their study, the predicted 4-week mortality rate of our cohort was calculated, and we found that 29.6% of patients in the VEN-plus-AZA group had a >20% risk for early mortality if they received intensive chemotherapy as induction treatment. The 1-year OS rates of the VEN-plus-AZA and chemotherapy groups did not differ significantly (65.3% vs. 80.7%). The 1-year disease relapse rate did not significantly differ between the VEN-plus-AZA and chemotherapy groups (19.7% vs. 18.0%, *p*-value = 0.91). The VEN-plus-AZA group had a higher 1-year NRM than the chemotherapy group (27.4% vs. 5.9%, *p* = 0.053). A worse NRM might reflect the fact that the patients in the VEN-plus-AZA group were older and had more comorbidities. Based on the findings of the multivariate analysis, the CIs of NRM between the VEN-plus-AZA and chemotherapy groups did not differ significantly.

Low HCT-CI scores and good-intermediate cytogenetic risks were significantly associated with a superior OS based on the univariate and multivariate analyses. These data indicate that baseline genomic features and relevant comorbidities are more reliable predictors of HSCT outcomes than treatment intensity.

In this cohort, the percentage of undetectable flow-based MRD did not significantly differ between the VEN plus AZA and chemotherapy groups. The depth of response (CR vs. without CR) and the detection of flow-based MRD cannot determine the prognosis of post-transplant OS and disease relapse based on the univariate and multivariate analyses. Therefore, some studies have revealed that pre-transplant MRD negativity is associated with superior post-transplant outcomes [21,22]. However, other studies found that pre-transplant MRD negativity may not be an independent predictor of post-transplant OS in patients aged >60 years, especially those who received lower-intensity therapy as remission induction [22,23]. Thus, further studies should be performed to assess the impact of MRD negativity on post-transplant outcomes in patients with AML.

Nevertheless, this study had several limitations. First, the inherent bias of a relatively small-sized, short-follow-up, and retrospective study constituted a limitation. Due to the small sample size, the meta-analysis is notably underpowered, leading to inconclusive results. Second, not all patients routinely underwent examinations of all genes (such as *NPM1*) due to the lack of current reimbursement coverage in Taiwan. Third, not all patients in our cohort had their MRD performed at a reference laboratory. Therefore, we could only use clinical response to induction therapy rather than MRD in the multivariate analysis due to sample size limitations. The median post-HSCT follow-up period was short, and the long-term outcomes of the two groups were not examined.

Although it is a small and retrospective study, in older patients who are unfit for intensive chemotherapy, a low-intensity regimen with VEN plus AZA seems to be a suitable bridge therapy. Further, allo-HSCT is feasible and can be a curative option.

## Figures and Tables

**Table 1:** Characteristics of the study participants.

Variable	Chemotherapy Group (*n* = 34)	VEN + AZA Group (*n* = 27)	*p*-Value
Age, years Median (IQR)	40 (33–59)	65 (61–70)	<0.005
Sex	-	-	
Male	20 (58.8%)	15 (55.6%)	1.00
Female	14 (41.2%)	12 (44.4%)	
AML subtype			<0.005
De novo AML	30 (88.2%)	12 (44.4%)	
MDS-related AML	4 (11.8%)	15 (55.6%)	
Cytogenetic risk group			0.08
Good	8 (23.5%)	1 (3.7%)	
Intermediate	20 (58.8%)	18 (66.7%)	
Poor	6 (17.7%)	8 (29.6%)	
FLT3 ITD			0.37
Negative	21 (61.8%)	22 (81.0%)	
Positive	13 (38.2%)	5 (19.0%)	
Early mortality score for intensive chemotherapy			0.048
Low risk (0–4)	31 (91.2%)	19 (70.4%)	
High risk (>4)	3 (8.8%)	8 (29.6%)	
Clinical response to induction therapy before HSCT			0.11
CR	24 (70.6%)	13 (48.2%)	
CRi or MLFS	10 (29.4%)	14 (51.9%)	
Flowcytometry MRD before HSCT			1
Undetectable	22 (81.5%)	21 (77.8%)	
Positive	5 (18.5%)	6 (22.2%)	
Missing	7 (20.6%)	0 (0%)	
HCT-CI			0.02
0–1	27 (79.4%)	13 (48.1%)	
≥2	7 (20.6%)	14 (51.9%)	
HSCT type			0.72
MSD	2 (5.9%)	2 (7.4%)	
MUD	15 (44.1%)	9 (33.3%)	
Haplo	17 (50.0%)	16 (59.3%)	
CMV status			0.26
R+/D+	21 (61.8%)	22 (81.5%)	
R−/D−	1 (2.9%)	0 (0.0%)	
R−/D+	0 (0.0%)	0 (0.0%)	
R+/D−	6 (17.6%)	2 (7.4%)	
Missing	6 (17.6%)	3 (11.1%)	
Conditioning regimen			0.11
MAC	16 (47.1%)	7 (25.9%)	
RIC	18 (52.9%)	20 (74.1%)	

Abbreviations: AZA: azacitidine; AML: acute myeloid leukemia; CR: complete remission; CRi: complete remission with incomplete recovery; CMV: cytomegalovirus; D: donor; FLT3 ITD: FLT3 internal tandem duplication; Haplo: haploidentical; HCT-CI: hematopoietic cell transplantation comorbidity Index; HSCT: hematopoietic stem cell transplantation; MDS: myelodysplastic syndrome; MAC: myeloablative conditioning regimen; MLFS: morphologic leukemia-free state; MRD: minimal residual disease; MSD: matched sibling donor; MUD: matched unrelated donor; R: recipient; RIC: reduced-intensity conditioning regimen; VEN: venetoclax.

**Table 2:** Infectious events between the chemotherapy and VEN + AZA groups.

Variable	Chemotherapy Group (*n* = 34)	VEN + AZA Group (*n* = 27)
Infections within 30 days, %	21%	30%
Bacterial infection, *n*	6	8
Viral infection, *n*	1	0
EBV reactivation, *n* (%)	16 (47.1%)	17 (63.0%)
MSD, *n*	1	0
MUD, *n*	6	7
Haplo, *n*	9	10
PTLD, *n*	1	1
CMV reactivation, *n* (%)	18 (52.9%)	14 (51.9%)
CMV disease, *n* (%)	3 (8.8%)	0 (0%)

Abbreviations: CMV: cytomegalovirus; EBV: Epstein–Barr virus; MSD: matched sibling donor; MUD: matched unrelated donor; Haplo: haploidentical; PTLD: post-transplant lymphoproliferative disorders.

**Table 3:** Univariate analysis of overall survival, relapse, and NRM.

Variable	1-Year Overall Survival Rate (95% CI)	*p*-Value	1-Year Cumulative Incidence of Relapse (95% CI)	*p*-Value	1-Year Cumulative Incidence of NRM (95% CI)	*p*-Value
Age (years)	-	0.10		0.55		0.07
<60	83.6% (64.7–92.9%)		15.5% (5.50–30.2%)		6.10% (1.0–17.9%)	
≥60	61.7% (40.3–77.3%)		23.4% (9.1–41.3%)		26.7% (11.4–44.8%)	
AML subtype		0.22		0.13		0.67
De novo AML	77.1% (60.6–87.4%)		9.8% (3.0–21.2%)		11.4% (2.8–27.0%)	
MDS-related AML	67.0% (40.4–83.8%)		28.0% (9.4–50.3%)		18.2% (7.2–33.1%)	
Cytogenetic risks		0.03		0.68		0.16
Good-intermediate risk	80.4% (65.7–89.3%)		15.1% (6.6–27.0%)		12.9% (5.2–24.3%)	
Poor risk	51.1% (21.6–74.5%)		30.5% (8.5–56.5%)		22.7% (4.9–48.3%)	
Flt3 mutation status		0.90		0.02		0.14
Without mutation	72.2% (55.2–83.6%)		14.7% (5.8–27.5%)		19.4% (9.0–32.9%)	
With mutation	77.8% (51.1–91.0%)		27.8% (9.6–49.6%)		5.6% (0.3–23.1%)	
Clinical response to induction therapy before HSCT		0.34		0.54		0.19
CR	75.9% (57.2–87.2%)		19.9% (8.6–34.6%)		11.3% (3.5–24.2%)	
CRi or MLFS	70.4%% (47.7–84.7%)		17.2% (5.1–35.1%)		21.1% (7.4–39.4%)	
Flowcytometry MRD before HSCT		0.57		0.72		0.89
Undetectable	74.8% (58.1–85.6%)		17.0% (7.4–30.0%)		17.1% (7.4–30.2%)	
Positive	63.6% (29.7–84.5%)		27.3% (5.7–55.4%)		18.2% (2.4–45.9%)	
HCT-CI		0.02		0.58		0.22
0–1	82.0% (65.9–91.0%)		15.0% (6.0–27.8%)		12.6% (4.5–25%)	
≥2	58.3% (33.4–76.7%)		27.3% (9.3–49.2%)		20.1% (6.0–40.0%)	
HSCT type		0.22		0.37		0.67
Matched donor	83.7% (62.2–93.6%)		15% (4.6–31.1%)		11.4% (2.8–27.0%)	
Haplo	66.0% (40.4–83.8%)		21.7% (9.3–37.3%)		18.2% (7.2–33.1%)	
Conditioning regimen		0.8		0.04		0.77
MAC	78.3% (55.4–90.3%)		4.3% (0.3–18.7%)		17.4% (5.2–35.4%)	
RIC	71.6% (53.6–83.7%)		27.4% (14.0–42.5%)		13.7% (4.9–27.0%)	

Abbreviations: CR: complete remission; CRi: complete remission with incomplete recovery; CI: confidence interval; FLT3 ITD: FLT3 internal tandem duplication; Haplo: haploidentical; HCT-CI: hematopoietic cell transplantation comorbidity Index; HSCT: hematopoietic stem cell transplantation; MDS: myelodysplastic syndrome; MAC: myeloablative conditioning regimen; MLFS: morphologic leukemia-free state; MRD: minimal residual disease; NRM: non-relapse mortality; RIC: reduced-intensity conditioning regimen; VEN: venetoclax.

**Table 4:** Univariate analysis of acute GVHD and chronic GVHd.

Variable	100-Day Cumulative Incidence of aGVHD (95% CI)	*p*-Value	1-Year Cumulative Incidence of cGVHD (95% CI)	*p*-Value
Age (years)	-	0.93	-	0.18
<60	18.2% (7.2–33.1%)		49.6% (23.4–57.1%)	
≥60	22.0% (8.7–39.1%)		27.0% (11.4–45.4%)	
AML subtype		0.25		0.75
De novo AML	14.4% (5.8–26.9%)		35.5% (20.8–50.4%)	
MDS-related AML	31.6% (12.4–52.9%)		31.6% (12.3–53.1%)	
Cytogenetic risks		0.95		0.37
Good-intermediate risk	19.1% (9.4–31.5%)		37.1% (23.1–51.1%)	
Poor risk	23.2% (5.1–48.8%)		25.5% (5.3–52.9%)	
Flt3 mutation status		0.23		0.75
Without mutation	23.7% (12.2–37.5%)		34.5% (20.2–49.2%)	
With mutation	11.1% (1.7–34.4%)		34.0% (13.1–56.4%)	
Clinical response to induction therapy before HSCT		0.27		0.74
CR	16.5% (6.6–30.4%)		31.5% (16.9–47.2%)	
CRi or MLFS	25.0% (9.9–43.6%)		38.5% (18.8–57.9%)	
Flowcytometry MRD before HSCT		0.23		0.75
Undetectable	23.7% (12.2–37.5%)		34.5% (20.2–49.2%)	
Positive	11.1% (1.7–30.4%)		34.0% (13.1–56.4%)	
HCT-CI		0.12		0.69
0–1	15.0% (6.0–27.8%)		30.7% (17.0–45.5%)	
≥2	29.8% (11.7–50.5%)		43.3% (19.2–65.4%)	
HSCT type		0.95		0.15
Matched donor	18.4% (6.5–35.0%)		42.6% (23.1–61.0%)	
Haplo	21.2% (9.2–36.5%)		27.9% (13.6–44.3%)	
Conditioning regimen		0.23		0.27
MAC	13.0% (3.1–30.2%)		26.1% (10.3–45.2%)	
RIC	24.2% (11.9–38.9%)		39.7% (23.4–55.7%)	

Abbreviations: aGVHD: acute graft-versus-host disease; cGVHD: chronic graft-versus-host disease; CR: complete remission; CRi: complete remission with incomplete recovery; CI: confidence interval; Haplo: haploidentical; HCT-CI: hematopoietic cell transplantation comorbidity Index; HSCT: hematopoietic stem cell transplantation; MDS: myelodysplastic syndrome; MAC: myeloablative conditioning regimen; MLFS: morphologic leukemia-free state; MRD: minimal residual disease; RIC: reduced-intensity conditioning regimen.

## Data Availability

The data are available upon reasonable request from the authors.

## Supplementary Materials

The following supporting information can be downloaded at: https://www.mdpi.com/article/10.3390/cancers16061082/s1, Figure S1: Forest plot of the multivariate analysis of overall survival; Figure S2: Forest plot of the multivariate analysis of disease relapse; Figure S3: Forest plot of the multivariate analysis of nonrelapse mortality; Figure S4: Forest plot of the multivariate analysis of acute GVHD; Figure S5: Forest plot of the multivariate analysis of chronic GVHD.
