# Supplementary material for: Venetoclax Plus Azacitidine as a Bridge Treatment to Allogeneic Stem Cell Transplantation in Unfit Patients with Acute Myeloid Leukemia
Source: Cancers (Basel). 2024 Mar 7;16(6):1082. doi: 10.3390/cancers16061082 (PMC10968407; doi:10.3390/cancers16061082)
Supplement: Supplementary file 1 [file cancers-16-01082-s001.zip › cancers-2871110-supplementary.pdf]

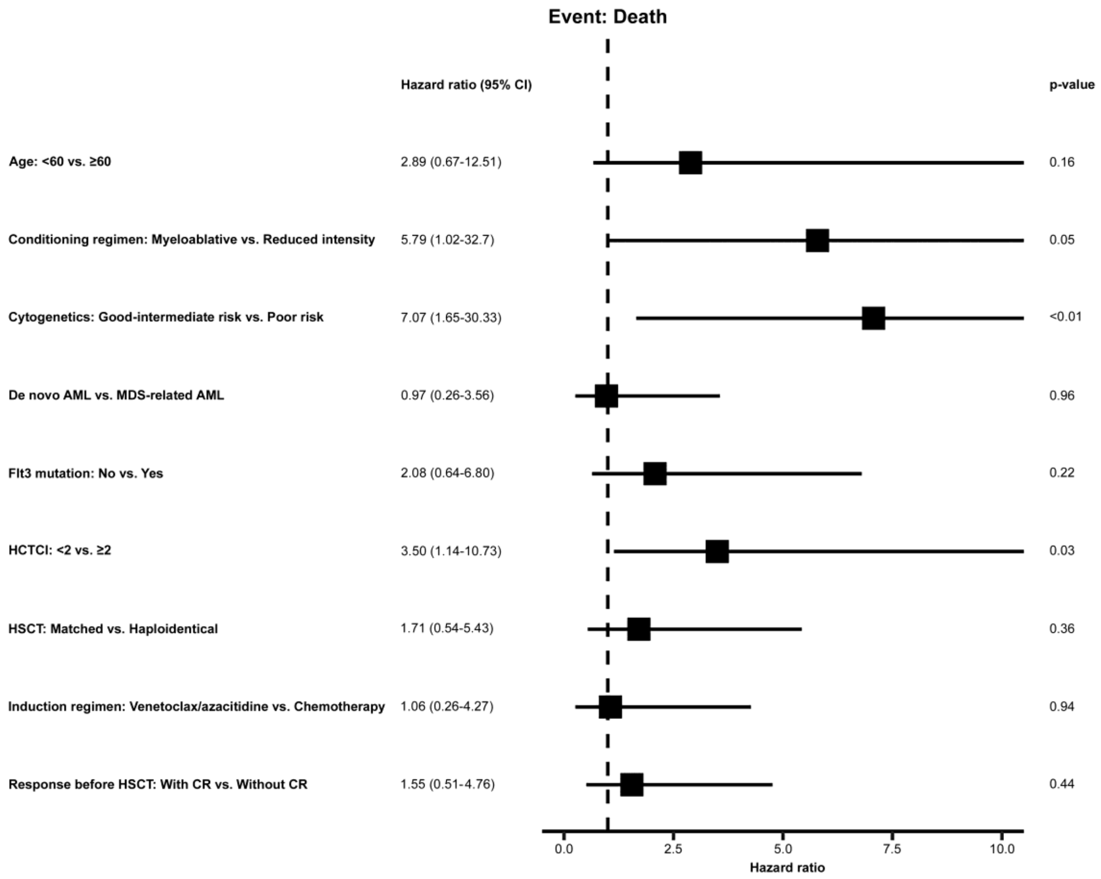

Supplemental Figure S1. Forest plot of the multivariate analysis of overall survival. On the left side, the variables are listed, alongside their adjusted hazard ratios with 95% confidence intervals. On the right side, the respective P-values are reported. CI, confidence interval.

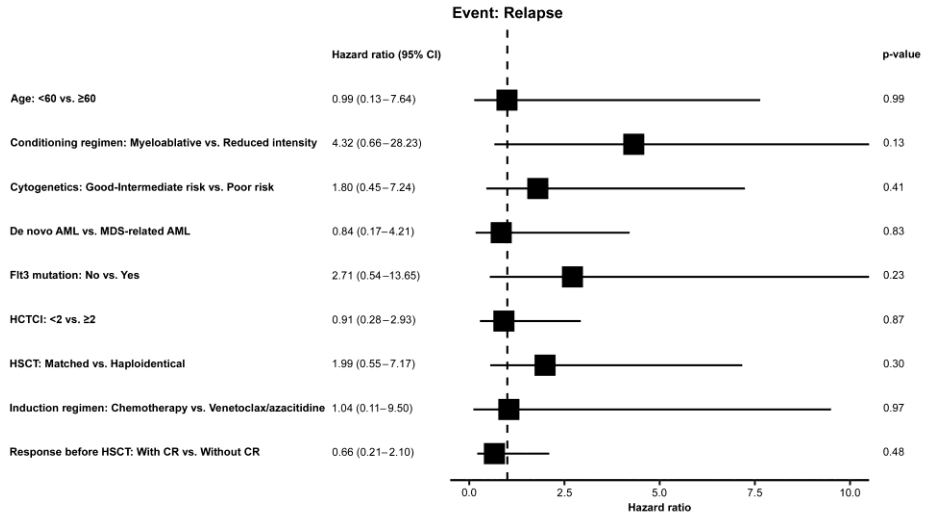

Supplemental Figure S2. Forest plot of the multivariate analysis of disease relapse. On the left side, the variables are listed, alongside their adjusted hazard ratios with 95% confidence intervals. On the right side, the respective P-values are reported. CI, confidence interval.

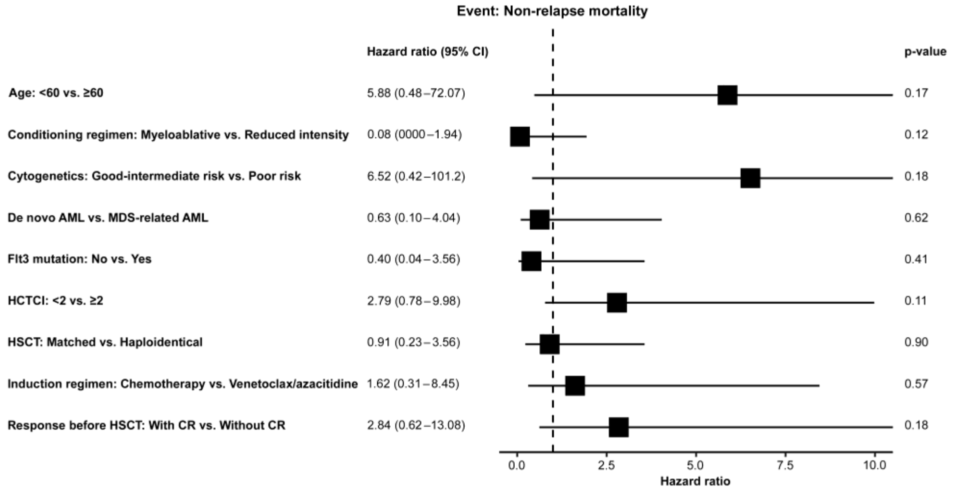

Supplemental Figure S3. Forest plot of the multivariate analysis of nonrelapse mortality. On the left side, the variables are listed, alongside their adjusted hazard ratios with 95% confidence intervals. On the right side, the respective P-values are reported. CI, confidence interval.

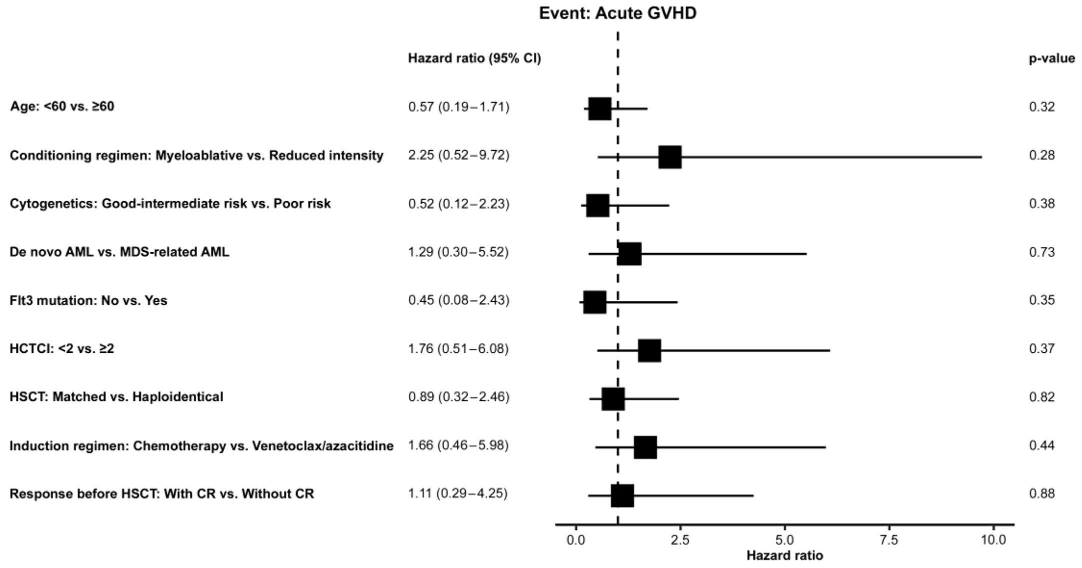

Supplemental Figure S4. Forest plot of the multivariate analysis of acute GVHD. On the left side, the variables are listed, alongside their adjusted hazard ratios with 95% confidence intervals. On the right side, the respective P-values are reported. CI, confidence interval.

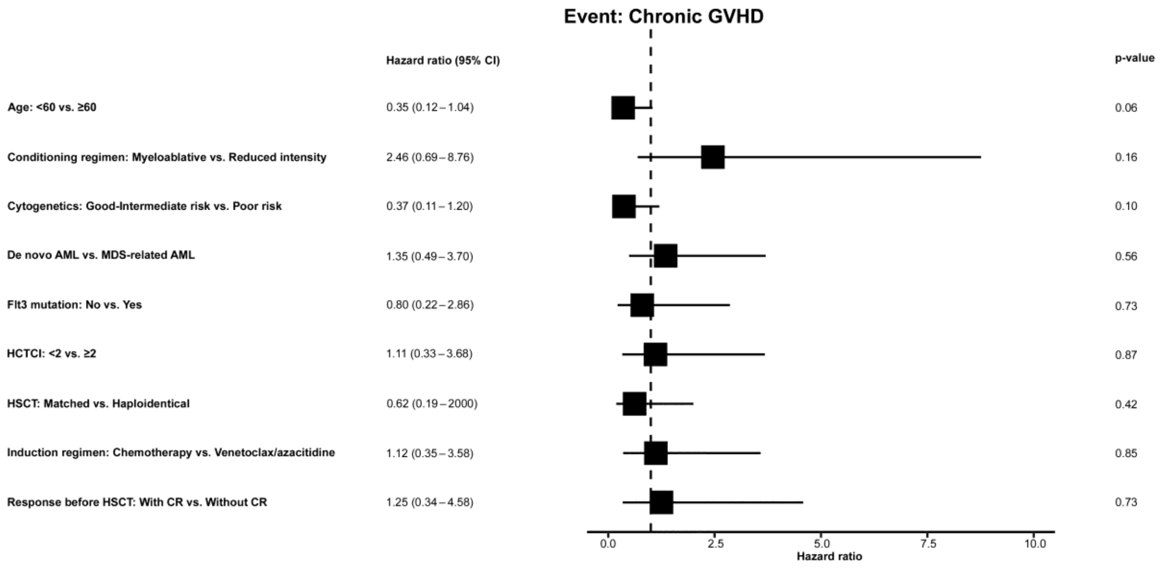

Supplemental Figure S5. Forest plot of the multivariate analysis of chronic GVHD. On the left side, the variables are listed, alongside their adjusted hazard ratios with 95% confidence intervals. On the right side, the respective P-values are reported. CI, confidence interval.
